# A Clinical Quality-Assurance Panel for Patient-Facing Black-Box Large Language Models in Healthcare: Anchored Forced-Choice Drift Monitoring for Sensitive Communication

**DOI:** 10.7759/cureus.108195

**Published:** 2026-05-03

**Authors:** Yuusuke Harada

**Affiliations:** 1 Graduate School of Public Policy, Hosei University, Tokyo, JPN; 2 Graduate School of Humanities and Social Sciences, Hiroshima University, Hiroshima, JPN

**Keywords:** black box, clinical laboratory quality assurance, large language models, monitoring, two-alternative forced-choice

## Abstract

Background

Patient-facing large language models (LLMs) are being integrated into patient portals, automated health messaging, psychoeducation, and mental health-related support. Because many deployments are vendor-hosted black boxes that may change without local visibility, health systems need practical quality-assurance methods that can establish a baseline behavioral signature and flag candidate changes in how sensitive topics are framed.

Methods

This feasibility study applied an anchored two-alternative forced-choice (2AFC) protocol to ChatGPT 5.4 (OpenAI, San Francisco, California, United States) through its consumer-facing interface on 25 March 2026. The primary objective was to determine whether a version-controlled anchored 2AFC panel could produce a parsable, repeatable, clinically interpretable baseline signature for a black-box LLM under a fixed prompt family. Secondary objectives were to estimate an anchored scale, place selected clinically salient concepts on that scale, quantify repeatability and confidence behavior, and explore sensitivity to wrapper and prompt-family changes. On each of 190 trials, the model compared two same-sized “solids” and chose which was harder (resistance to indentation and scratching; brittleness excluded), then returned a one-line JavaScript Object Notation (JSON) object containing a forced-choice response and a confidence score (0-100). A Bradley-Terry model generated an anchored 0-100 hardness scale (Marshmallow = 0; Steel = 100) with parametric bootstrap confidence intervals (CIs). We also executed the same panel on a locally hosted model to assess on-premise feasibility.

Results

The anchored panel produced a coherent within-session baseline signature with complete repeated-pair agreement (all 64 repeated unordered pairs yielded identical winners) and near-perfect Bradley-Terry fit (classification accuracy 1.000; log loss 0.0117). The scale separated lower-hardness concepts (Life 6.04, 95% CI -0.79 to 8.69; Kindness 13.70, 95% CI 4.42 to 16.79; Silence 21.14, 95% CI 12.34 to 24.09) from higher-hardness concepts (Justice 75.93, 95% CI 71.59 to 83.91; Death 87.15, 95% CI 84.59 to 88.31). Model-reported confidence correlated strongly with inferred pair distance (r = 0.842, 95% CI 0.813-0.869). Style/language wrappers showed complete agreement, whereas a prompt-family comparison produced three reversals among 36 hardness pairs. In the local-model feasibility run, internal consistency was lower (accuracy 0.926; log loss 0.158) with anchor-order violations (Polycarbonate sheet > Steel), demonstrating that the panel can flag deviations that warrant clinical or governance review.

Conclusions

Anchored forced-choice panels can serve as compact, repeatable behavioral control materials for patient-facing LLM governance by establishing a baseline for later regression testing, post-update monitoring, and prompt-template change control. This single-model, single-session feasibility study does not demonstrate longitudinal drift detection, clinical outcomes, or therapeutic appropriateness. The instrument should therefore complement, not replace, scenario-based clinical safety evaluation, expert review, and organizational oversight.

## Introduction

Patient-facing large language models (LLMs) are rapidly moving from demonstrations to real-world use in patient portals, discharge instructions, automated triage, psychoeducation, and mental health-related conversational support [1-4]. In clinical practice, the risk is not only that a model can be wrong, but that its communication style, risk framing, and safety-escalation behavior can change after deployment, particularly when vendors update models or when local prompt templates change [5,6]. For clinical teams, this creates a practical safety problem: a tool that was judged acceptable during procurement or pilot testing may not retain the same behavioral profile when patients encounter it later.

In many deployments, the model is operationally a black box. Local teams can observe input-output behavior but cannot inspect model internals, control decoding settings, or reliably detect silent version changes. This creates a governance gap for clinicians and health systems: even if pre-deployment testing is performed, there is no guarantee that the same behavioral profile persists weeks later. Behavioral black-box testing, therefore, remains a realistic way to generate actionable evidence under operational constraints [7-10].

Clinical medicine already uses continuous quality-assurance paradigms when a process is safety-critical. Laboratories run control materials, imaging services calibrate against phantoms, and clinical programs use audit-and-feedback loops to detect drift and trigger review. Patient-facing LLMs that participate in healthcare communication should be treated similarly: health systems need low-burden, repeatable sentinel measurements that can be rerun on demand, compared against baselines, and embedded into change-control workflows. From a patient-safety perspective, drift in how an LLM frames sensitive topics - such as death, justice, coercion, or self-harm - may affect trust, crisis communication, and escalation pathways even when factual accuracy appears unchanged [3,5,6].

We propose an anchored forced-choice panel as a pragmatic quality-assurance assay for this governance problem. Pairwise forced-choice designs are attractive in clinical quality assurance because they are simple, machine-readable, and amenable to statistical summaries such as repeatability and confidence intervals (CIs). We use the Bradley-Terry paired-comparison framework [11] and an anchoring strategy that fixes scale endpoints, analogous to calibration points in clinical measurement. In this feasibility study, we use a hardness metaphor to create a stable anchored axis; however, the larger contribution is the operational framework: a version-controlled panel that yields a reproducible behavioral signature suitable for baseline documentation and later drift monitoring. Because prompt wording and wrapper style can materially affect model behavior, the prompt family must be treated as part of the measurement instrument and governed accordingly [12-14].

The primary objective of this study was to determine whether a version-controlled anchored 2AFC panel could produce a usable, repeatable baseline behavioral signature for a black-box, patient-facing LLM in the same access environment used by clinicians or patients. Secondary objectives were to (1) construct an anchored forced-choice scale using physical materials as reference controls, (2) place a small set of clinically salient concepts on that scale with uncertainty estimates, (3) quantify within-session repeated-pair agreement, Bradley-Terry model fit, and the confidence-distance association, (4) evaluate sensitivity to superficial wrapper changes versus substantive prompt-family changes, and (5) demonstrate whether the same workflow can be executed on a locally hosted model. Feasibility was interpreted in measurement terms rather than clinical-efficacy terms: the panel would be considered useful if it produced machine-parseable outputs, preserved the intended anchor hierarchy in the primary run, showed high within-session repeatability, yielded an interpretable one-dimensional model fit, and flagged deviations under substantive prompt or model changes. These criteria do not establish clinical safety or patient benefit; they establish whether the proposed quality-control assay is operationally usable.

## Materials and methods

Study design and system under study

This was a computational quality-assurance study of a single proprietary LLM treated as a black-box system. No human participants, patient data, or protected health information were involved. All responses were collected from ChatGPT 5.4 (OpenAI, San Francisco, California, United States) through the consumer-facing ChatGPT interface on 25 March 2026. The interface did not expose a stable build identifier beyond the product label, and temperature and random seed were not user-controllable.

This access mode was deliberate. Because many healthcare professionals and patients encounter LLMs through consumer-facing interfaces, the proposed quality-control panel is designed to audit the same environment that end users actually experience. We therefore prioritized ecological validity at the point of use over the tighter parameter control available through an application programming interface (API). The trade-off is that parameter-level reproducibility is limited: the run can be operationally replicated, but not exactly reproduced with the same model build, decoding temperature, or random seed. Accordingly, reproducibility in this study refers to reproducing the documented panel, trial file, prompt family, and analytic pipeline, rather than guaranteeing identical response regeneration from an immutable proprietary model build.

Clinical objective and feasibility criteria

The clinical objective was not to evaluate diagnosis, treatment selection, or therapeutic communication quality directly. Instead, the panel was designed as a candidate post-deployment quality-assurance instrument for systems that generate patient-facing language. The intended governance use is to document a baseline behavioral signature under a defined prompt family and then rerun the same panel after a vendor update, local prompt revision, or safety incident.

For this feasibility study, the panel was judged against operational measurement criteria: (1) responses should be machine-parseable under the specified JSON schema; (2) reference materials should form an anchor-respecting scale in the primary run; (3) repeated unordered pairs should yield high agreement; (4) the Bradley-Terry model should summarize the observed pairwise choices with interpretable fit statistics; and (5) wrapper or prompt-family changes should be measurable as either invariance or reversals. These criteria are quality-control criteria, not patient-outcome criteria.

Anchored two-alternative forced-choice (2AFC) protocol

Each trial presented two labeled items (A and B) and asked the model to imagine them as same-sized solid materials. The task was to choose which item was harder, where hardness was defined as resistance to indentation and scratching, and brittleness was excluded. The model also reported an integer confidence rating from 0 to 100. Outputs were restricted to a one-line JSON object containing trial_id, choice, and confidence to support automated parsing and repeated re-administration as a governance assay.

The prompt family, item labels, trial identifiers, A/B ordering, and output schema were treated as part of the measurement instrument. The core hardness prompt template used the following structure, with trial-specific identifiers and labels inserted programmatically or manually into the bracketed fields:

Quality-assurance forced-choice trial. Compare A and B as same-sized solid materials. Hardness means resistance to indentation and scratching. Exclude brittleness, fragility, size, weight, emotional valence, moral desirability, and clinical importance. Trial_id: [TRIAL_ID]. A: [ITEM_A]. B: [ITEM_B]. Choose the harder item. Return exactly one line of JSON and no other text: {"trial_id":"[TRIAL_ID]","choice":"A" or "B","confidence":[integer 0-100]}.

The full prompt and run-documentation template are provided in Appendix 1.

Wrapper-invariance prompts altered the surrounding style or language while preserving the forced-choice task, the item labels, and the JSON schema. Substantive prompt-family changes were defined as changes that altered the broader framing of the comparison task rather than merely changing language or tone. In deployment, the complete 190-row run file, including trial order and A/B position, should be archived with the prompt template and raw JSON outputs because these elements determine whether longitudinal comparisons are meaningful.

Stimuli and clinical rationale for target concepts

Ten material anchors were selected to span an intuitive hardness continuum: Marshmallow, Oak hardwood, Acrylonitrile butadiene styrene (ABS) plastic block, Polycarbonate sheet, Acrylic sheet, Aluminum plate, Dry bone, Window glass, Porcelain, and Steel. Five abstract concepts were selected as targets: Life, Kindness, Silence, Justice, and Death.

Although abstract, these targets are frequently encountered in clinically consequential communication domains such as palliative care and bereavement (death), trauma-informed care (silence), ethics and procedural justice (justice), and supportive communication (kindness, life). In the present study, they serve as a feasibility set to demonstrate anchored scaling, repeatability, and invariance testing. Importantly, the panel is intended as an instrument template: health systems can extend the item set to more directly clinical vocabularies (e.g., self-harm, safety planning, consent, confidentiality) when building deployment-specific governance panels.

Experimental design and statistical analysis

The main experiment comprised 190 trials, including concept-anchor comparisons, concept-concept comparisons, midrange anchor-anchor comparisons, and catch trials. Pairwise choices were modeled with the Bradley-Terry framework [11]. Each trial was encoded as a paired-comparison observation, and latent item parameters were estimated with L2-regularized logistic regression. Fitted scores were then linearly rescaled so that Marshmallow = 0 and Steel = 100. Ninety-five percent confidence intervals (CIs) were estimated via parametric bootstrap [15,16] by simulating outcomes from the fitted Bradley-Terry probabilities, refitting the model for each replicate, and taking percentile bounds on the anchored scale. Model fit was summarized with classification accuracy and log loss against the observed winners.

**Figure 1:**
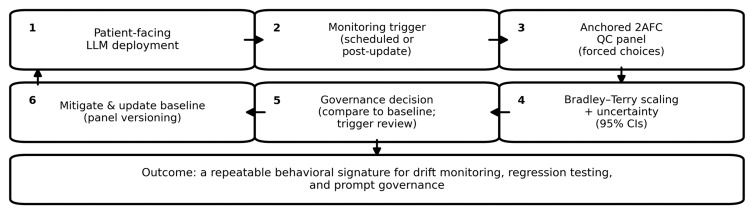
Clinical governance workflow for an anchored two-alternative forced-choice (2AFC) quality-control (QC) panel. At scheduled intervals - or after a vendor/model update - the panel is rerun, Bradley-Terry estimates with uncertainty are compared with the stored baseline, and predefined thresholds trigger clinical review, mitigation, or baseline versioning. LLM: large language model

Within-session repeatability was summarized as agreement in the inferred winner across repeated unordered pairs. The confidence-distance association was quantified as the correlation coefficient (r) between reported confidence and the absolute difference between the two anchored hardness scores. Prompt sensitivity was explored descriptively via (1) a style/language wrapper invariance pilot and (2) a prompt-family comparison, analogous to measurement-invariance checks in psychometrics [17-19]. All statistical analyses were performed in Python using custom scripts (Python Software Foundation, Wilmington, Delaware, United States). The proposed clinical governance workflow is summarized in Figure 1.

The locally hosted model was included as an on-premise feasibility example rather than as a controlled benchmark against the proprietary system. Accordingly, the local-model results are interpreted as a demonstration that the same black-box panel can surface quality-control flags in a different deployment environment, not as evidence that one model is superior to another. For future deployment-specific replication, local model identifier, parameter count, quantization state, decoding settings, hardware environment, prompt template, run file, and raw outputs should be logged with the panel version.

## Results

**Figure 2:**
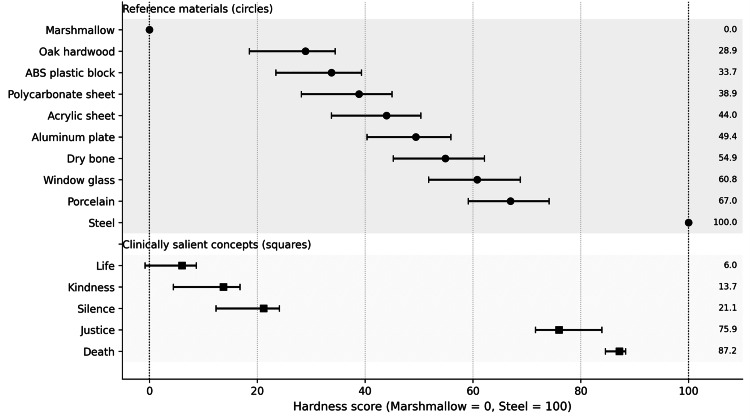
Anchored hardness scale for reference materials and concept targets. Points denote Bradley-Terry estimates; horizontal bars denote 95% bootstrap CIs.

As a governance assay, the anchored panel produced a coherent reference-material scale and enabled placement of a small set of clinically salient concepts with uncertainty estimates. Figure 2 shows the combined anchor and concept placements on the same 0-100 anchored scale, and Table 1 reports the corresponding point estimates and bootstrap intervals.

**Table 1:** Anchored hardness values for reference materials and concept targets (0–100 scale). ABS: acrylonitrile butadiene styrene; CI: confidence interval.

Item	Type	Hardness (0–100)	95% CI (low–high)
Marshmallow	Anchor material	0.00	0.00–0.00
Life	Concept (target)	6.04	-0.79–8.69
Kindness	Concept (target)	13.70	4.42–16.79
Silence	Concept (target)	21.14	12.34–24.09
Oak hardwood	Anchor material	28.93	18.53–34.48
ABS plastic block	Anchor material	33.74	23.47–39.32
Polycarbonate sheet	Anchor material	38.88	28.18–45.00
Acrylic sheet	Anchor material	43.95	33.74–50.33
Aluminum plate	Anchor material	49.38	40.38–55.89
Dry bone	Anchor material	54.89	45.23–62.16
Window glass	Anchor material	60.77	51.81–68.79
Porcelain	Anchor material	66.95	59.16–74.11
Justice	Concept (target)	75.93	71.59–83.91
Death	Concept (target)	87.15	84.59–88.31
Steel	Anchor material	100.00	100.00–100.00

Model fit, repeatability, and confidence behavior

The one-dimensional Bradley-Terry model reproduced the observed pairwise judgments extremely well (classification accuracy 1.000; log loss 0.0117). Within-session repeatability was complete: all 64 repeated unordered pairs yielded identical winners. Reported confidence tracked inferred pair distance strongly (r = 0.842, 95% CI 0.813-0.869), consistent with confidence behaving as a within-task margin signal rather than as a calibrated probability [20]. Key model-fit and repeatability metrics are summarized in Table 2.

**Table 2:** Summary metrics for the anchored two-alternative forced-choice (2AFC) panel.

Metric	Value
Bradley-Terry classification accuracy	1.000
Bradley-Terry log loss	0.0117
Repeated-pair agreement (n=64 pairs)	100% (all winners identical across repetitions)
Confidence vs absolute hardness difference	r = 0.842 (95% CI 0.813–0.869)

Prompt sensitivity and invariance checks

**Figure 3:**
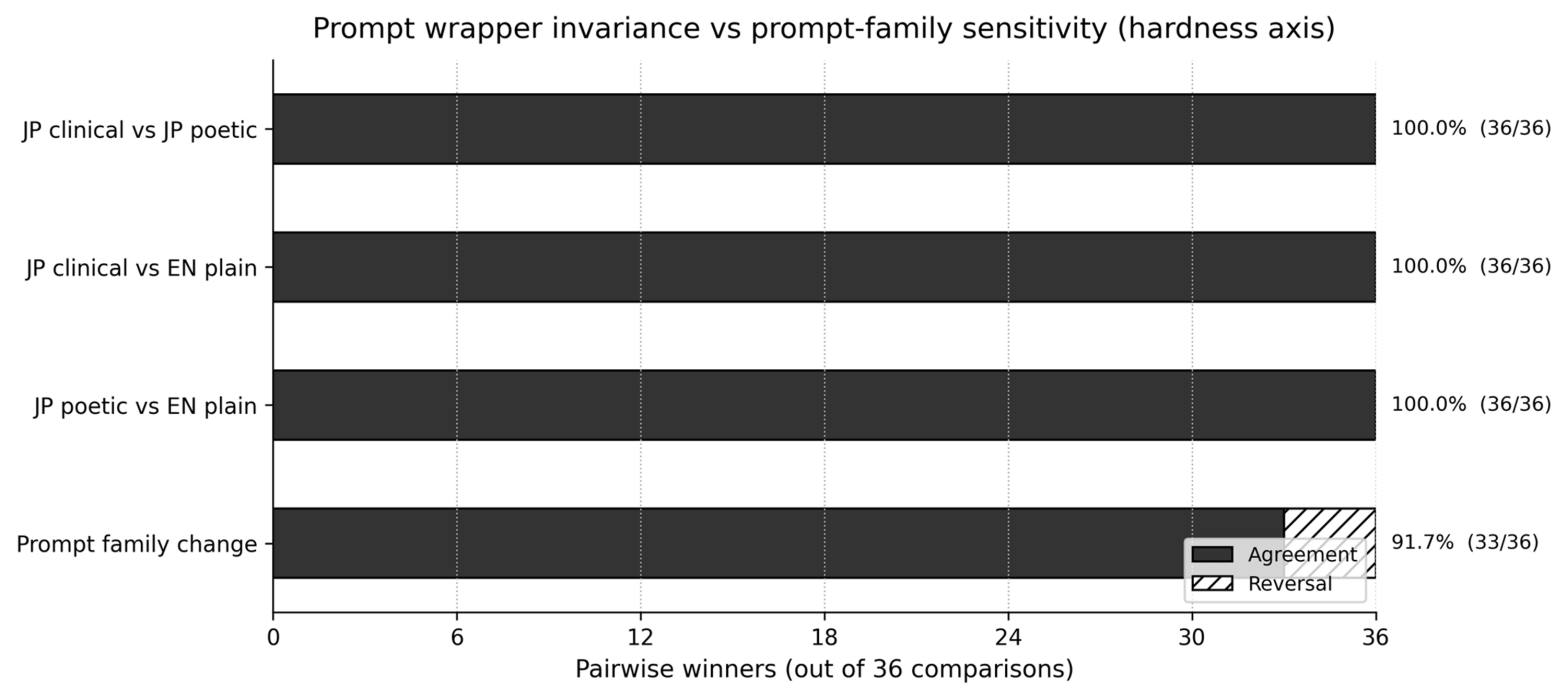
Pairwise agreement under wrapper invariance and prompt-family change (hardness axis). JP: Japanese; EN: English

A wrapper invariance pilot compared Japanese clinical, Japanese poetic, and English plain prompt wrappers under a fixed item set. All wrapper comparisons yielded identical pairwise winners (100% agreement). In contrast, changing prompt families yielded three reversals among 36 hardness pairs (91.7% agreement), indicating that the prompt family functions as the measurement instrument and should be version-controlled in governance workflows [17-19]. Results are summarized in Figure 3 and Table 3.

**Table 3:** Pairwise agreement in wrapper invariance and prompt-family sensitivity comparisons. HARD: hardness axis; TEMP: temperature axis.

Axis	Comparison	Unordered pairs (n)	Reversals (n)	Agreement (%)
HARD	Japanese clinical vs Japanese poetic	36	0	100.0
HARD	Japanese clinical vs English plain	36	0	100.0
HARD	Japanese poetic vs English plain	36	0	100.0
HARD	Baseline prompt family vs wrapper family	36	3	91.7
TEMP	Japanese clinical vs Japanese poetic	28	0	100.0
TEMP	Japanese clinical vs English plain	28	0	100.0
TEMP	Japanese poetic vs English plain	28	0	100.0

Exploratory two-axis pilot

**Figure 4:**
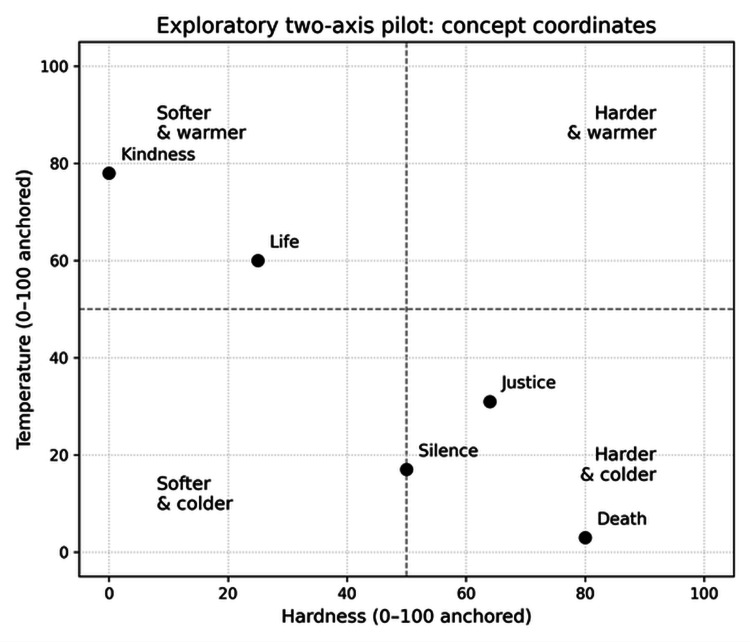
Exploratory two-axis pilot mapping concepts in hardness–temperature space (axes anchored independently).

To illustrate extensibility beyond a single sensory dimension, an earlier pilot mapped the same five concepts onto both hardness and temperature axes. Figure 4 shows the concept coordinates in hardness-temperature space with each axis anchored independently. Corresponding numerical coordinates are provided in Table 4.

**Table 4:** Exploratory two-axis pilot: concept coordinates in hardness–temperature space.

Concept	Hardness (0–100)	Temperature (0–100)
Life	25.17	60.29
Kindness	0.62	77.72
Silence	49.97	17.24
Justice	63.77	30.87
Death	79.63	2.99

Local-model replication pilot (on-premise feasibility)

**Figure 5:**
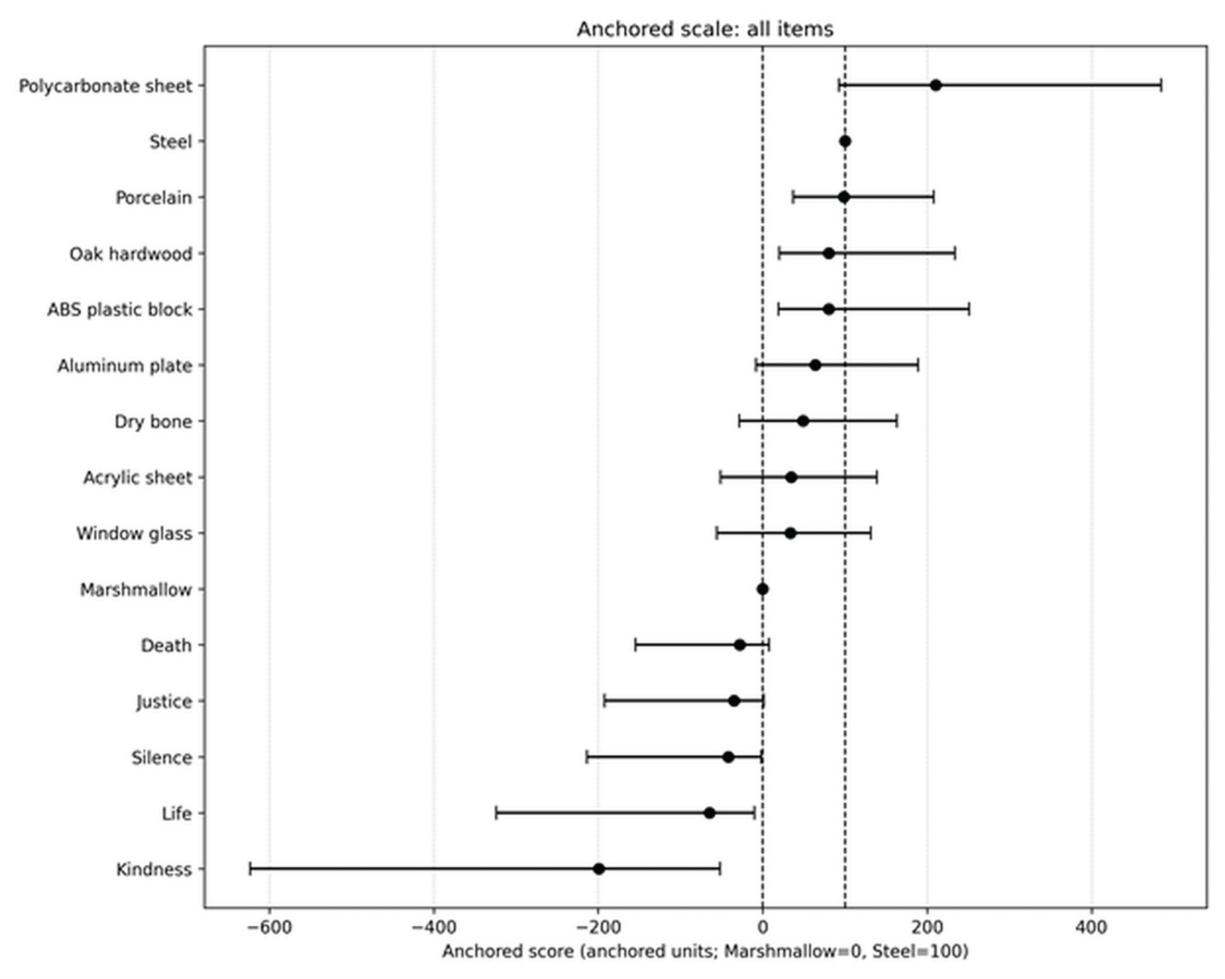
Anchored scale for the locally hosted model across all panel items. Scores are in anchored units (marshmallow = 0; steel = 100); items may fall outside the anchor range when ranked beyond the anchors. The pattern shows anchor-order violations (polycarbonate sheet > steel) and abstract concept targets below marshmallow, triggering a quality-control flag before patient-facing use.

**Figure 6:**
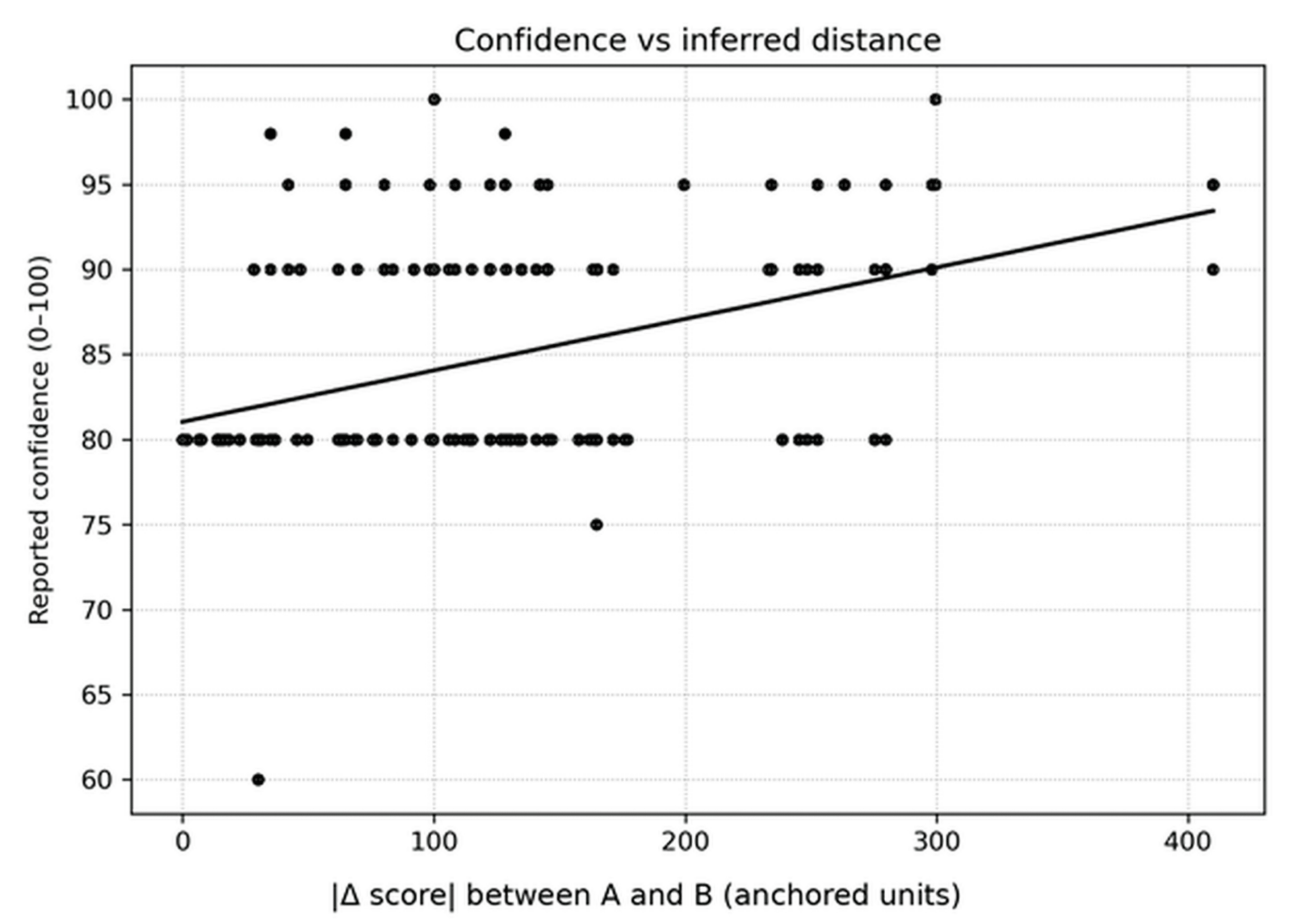
Relationship between model-reported confidence and inferred distance between compared items (absolute difference in anchored units) for the local-model replication. The attenuated slope and correlation (r = 0.413, 95% CI 0.298-0.521) relative to the primary run illustrate reduced internal calibration and support using the panel as a lightweight behavioral quality assurance (QA) instrument.

To demonstrate feasibility for health systems that deploy LLMs on-premise, we executed the identical anchored 2AFC panel on a locally hosted model (n=190). Quality-control metrics are summarized in Table 5. Relative to the primary black-box run (Table 2), overall fit remained high (accuracy=0.926; log loss=0.158) but repeatability decreased (repeat agreement=0.906), the confidence-distance association weakened (r=0.413, 95% CI 0.298-0.521), and anchor-order violations increased (1/8). Figure 5 visualizes the resulting anchored signature across all items, and Figure 6 shows the attenuated confidence-distance relationship. These findings are interpreted as an operational quality-control flag rather than as a formal benchmark of local-model quality.

**Table 5:** Local-model replication quality-control summary. QC: quality control

Metric	Value
Bradley-Terry classification accuracy	0.926
Bradley-Terry log loss	0.158
Repeated-pair agreement (n=64 pairs)	90.6% (58/64)
Confidence vs absolute hardness difference	r = 0.413 (95% CI 0.298–0.521)
Anchor-order sanity check (illustrative)	Polycarbonate sheet > Steel; concepts < Marshmallow (QC flag)

## Discussion

Principal findings

This feasibility study shows that a patient-facing black-box LLM can be instrumented with an anchored 2AFC panel and summarized with quantities that are operationally familiar in clinical quality assurance: point estimates with uncertainty, repeatability, and invariance checks. In our main experiment, the recovered ordering was coherent, the model fit was near-perfect, repeated-pair agreement was complete, and confidence scaled with inferred pair distance. Together, these features support the view that a version-controlled prompt family can elicit a measurable baseline response policy that can be compared across future runs.

The results also show why the prompt should be treated as part of the measurement instrument. Superficial wrapper changes preserved pairwise winners in this pilot, whereas a broader prompt-family change produced detectable reversals. The locally hosted model feasibility run produced lower repeatability, weaker confidence-distance association, and anchor-order violations, illustrating how the same panel can flag behavior that warrants review in a different access environment.

What this study does and does not show

The present data support the feasibility of baseline measurement, not proof of longitudinal drift detection. We did not rerun the same system across a known vendor update, nor did we link score changes to patient outcomes, communication quality, or adverse events. Therefore, the phrase “drift monitoring” should be understood as the intended governance use of the panel once a baseline is established and repeated over time, not as a completed longitudinal validation in this study.

Similarly, high internal consistency in this forced-choice task does not by itself establish that the model is clinically safe, empathic, or therapeutically appropriate. It shows that the model responded consistently to a defined measurement instrument. A clinically meaningful quality program would use this signal to trigger deeper evaluation, including scenario-based safety tests, clinician review, patient-facing usability assessment, and incident analysis.

Clinical contribution: post-deployment governance for patient-facing LLMs

The clinical value of this protocol is not diagnosis or treatment selection. Instead, it provides a lightweight post-deployment surveillance tool for health systems that deploy or permit patient-facing (or clinician-facing) LLMs for communication, psychoeducation, triage support, or mental health-related interactions. In such settings, silent vendor updates and local prompt-template changes create a practical change-management problem: the system that clinicians and patients encounter today may not match the one that was evaluated during procurement or pilot testing. An anchored forced-choice panel offers a compact behavioral “signature” that can be rerun after updates and compared against a documented baseline, analogous to running control materials in laboratory medicine.

From a patient-safety perspective, the concern is not limited to factual errors. Drift in how a model frames sensitive concepts can alter tone, perceived empathy, moral certainty, or the threshold for escalation in crisis-related dialogue. A small, repeatable panel cannot certify safety on its own, but it can provide an early warning signal that triggers targeted scenario-based testing and clinical review when behavioral shifts exceed a pre-specified tolerance band. This supports governance workflows such as acceptance testing, post-update regression testing, and incident review.

Anchored panels offer a concrete and scalable way to quantify qualitative shifts in a model’s patient-facing behavior while remaining interpretable to clinical stakeholders. This approach complements standard transparency artifacts (eg, Model Cards and Datasheets) by adding empirical, longitudinal behavioral measurement for a small set of high-salience concepts that matter in clinical communications [21,22].

Operationalizing the panel in a clinical setting

Operationalization can be simplified by treating panel runs as a recurring quality-assurance task assigned to a clinician champion or quality/safety lead, with a registry of baselines (panel versioning) and explicit change-control rules when drift is detected. Illustrative clinical governance uses are summarized in Table 6.

**Table 6:** Illustrative clinical governance uses of an anchored two-alternative forced-choice (2AFC) quality control panel. Synthesized from prior literature on regulatory oversight, behavioral testing, prompt effects, measurement invariance, and model documentation [5,6,9,13,14,17-19,21,22].

Governance activity	How the panel can be used	Clinical motivation
Pre-deployment acceptance testing	Run the panel on the intended access mode and prompt template; document baseline scores and CIs.	Establish a measurable baseline before patient exposure.
Post-update regression testing	Rerun the same panel after a vendor update or prompt change; quantify score shifts and reversals.	Detect silent drift that may alter sensitive framing.
Ongoing drift monitoring	Schedule periodic panel runs and track metrics over time (e.g., score changes vs baseline).	Support continuous oversight in black box environments.
Prompt-template governance	Treat the prompt family as the measurement instrument; require invariance checks when modifying wrappers or role prompts.	Avoid unintentional construct shifts caused by prompt redesign.
Incident review support	After a safety incident, rerun the panel to assess whether concept organization or confidence behavior has shifted.	Provide reproducible behavioral evidence for governance committees.

Construct validity of the hardness metaphor

A central limitation is that the hardness axis is an artificial metaphor, not an externally validated clinical construct. A concept’s placement on this scale should not be interpreted as a direct measure of empathy, risk, safety, or patient harm. The hardness axis is useful here because it provides an anchored, easily understood control dimension for testing whether a model’s organization of sensitive terms is stable under a fixed prompt instrument. Its clinical role is therefore sentinel detection: a change in concept placement or repeated-pair behavior would prompt review, but would not by itself prove that patient-facing communication has become unsafe.

Future panels should improve construct validity by using clinically anchored vocabularies and axes defined with clinical experts, such as urgency, escalation threshold, stigma, autonomy, empathy/tone, coercion sensitivity, or safety-planning orientation. Human expert ratings, patient-safety scenarios, and outcome-linked communication quality measures will be needed before score changes can be interpreted as clinically meaningful rather than merely measurement-relevant.

Measurement invariance, prompt governance, and clinical change control

The invariance pilot clarifies that the prompt family functions as the measurement instrument. In our pilot, superficial wrapper changes (including language shifts) preserved pairwise winners, whereas a broader prompt-family change produced detectable reversals. For clinical quality-control workflows, prompt templates should therefore be version-controlled, and invariance checks should accompany any substantive change in wrapper, language, or system role specification. Operationally, this mirrors change-control practice in clinical software: the instrument (here, the prompt family and item set) must be stable if longitudinal comparisons are to be meaningful.

Confidence behavior and monitoring design

The observed confidence-distance relationship suggests that the model’s self-reported confidence behaves like a within-task margin signal. However, model confidence should not be treated as a calibrated probability of correctness [20]. The high correlation in the primary run may partly reflect the structure of the forced-choice task and the separation of the anchored items. Confidence can still support monitoring design, for example, by concentrating additional pairwise comparisons near uncertain boundaries or concept regions that appear unstable after an update, but it should not be used as a standalone safety metric.

Limitations

Several limitations warrant emphasis. The central interpretive constraints are the artificial metaphor construct, the single-session cross-sectional design, limited parameter-level control in the consumer interface, and the small abstract vocabulary. First, the panel measures a prompt-defined metaphor axis rather than an external truth about human cognition, communication quality, or clinical outcomes; metaphor inference itself can be challenging and context sensitive in language models [12]. Second, the hardness axis is not clinically validated, and changes on this axis cannot be assumed to correspond to patient harm, empathy, safety escalation, or therapeutic appropriateness. Third, the study involved a single proprietary model accessed through a consumer interface without control over temperature or seed. This choice increases ecological validity for point-of-use governance but limits parameter-level reproducibility and generalizability.

Fourth, the study was single-session and cross-sectional. It established a baseline measurement procedure and examined prompt-family variation, but it did not empirically demonstrate longitudinal drift after a known vendor update. Fifth, the concept set was intentionally small and partly non-clinical; richer, deployment-specific vocabularies will be needed for high-stakes monitoring. Sixth, exact replication requires version-controlled prompt templates, trial order, A/B item positions, raw outputs, code, regularization settings, bootstrap settings, model access mode, and model identifiers when available. We archived the trial-level dataset and associated reproducibility materials under DOI: 10.5281/zenodo.19703796 to improve operational reproducibility; however, the consumer interface did not expose temperature, seed, or a stable build identifier, and these unavailable parameters continue to limit parameter-level reproducibility. Finally, the local-model replication was included to demonstrate on-premise feasibility and quality control (QC)-flag generation, not to provide a fair model benchmark; detailed architecture and decoding metadata would be required for any comparative model study.

Future directions toward clinically anchored panels

A priority for subsequent work is to extend the panel to clinically defined vocabularies and axes that map more directly onto patient-safety governance. Candidate targets include self-harm and suicidality language, abuse and coercion, stigma, consent and confidentiality, safety planning, intoxication, decisional capacity, and escalation instructions. Beyond a single axis, multi-axis panels could monitor separable dimensions such as urgency, empathy/tone, and risk-framing. Such expansions would allow the method to progress from feasibility toward clinically meaningful semantic surveillance, while retaining the operational advantages of a low-burden, repeatable black-box assay.

Future validation should include longitudinal reruns before and after known model or prompt-template changes, multiple proprietary and open models, controlled API experiments when available, expert-derived clinical anchors, and comparison with scenario-based patient-safety evaluations. Such work would clarify which panel shifts are merely technical signals and which predict meaningful changes in patient-facing communication.

## Conclusions

Anchored forced-choice scaling provides a compact, fully black-box method for quantifying how a patient-facing LLM organizes sensitive concepts under a defined, version-controlled prompt instrument. In this feasibility study, ChatGPT 5.4 yielded a stable anchored hardness signature, complete repeated-pair agreement, a strong confidence-distance relationship, and limited but detectable prompt-family sensitivity. For healthcare communication and mental health-related deployment, such panels can support post-deployment drift monitoring, regression testing after vendor updates, and prompt-template governance in the same consumer-facing environment that clinicians and patients encounter. Because this was a single-model, single-session feasibility study, the phrase drift monitoring denotes the intended post-deployment workflow of repeated comparison with a stored baseline, not an empirical demonstration of longitudinal drift after a known vendor update. These tools should be used alongside scenario-based clinical safety evaluation, expert review, and organizational oversight, not in place of them.

## Appendices

Appendix 1: Prompt and run-documentation template for replication

This appendix is included to address reproducibility and to support implementation as a quality-assurance instrument. The trial-level dataset and associated run materials are available under DOI: 10.5281/zenodo.19703796. In a clinical quality-assurance implementation, the same record set should be retained as institutional QC documentation whenever the panel is rerun or versioned.

**Table 7:** Version-controlled elements for replication and routine quality-assurance reruns.

Component	What should be version-controlled
Prompt family	Base instruction, wrapper instruction, language, role framing, and JSON schema
Item set	Exact spelling/capitalization of all anchors and concept targets
Trial file	trial_id, item_A, item_B, A/B order, trial block, repeat/catch indicator, and run order
Model access metadata	Access route, date/time, exposed model label/build identifier if available, temperature/seed if available
Raw outputs	Full one-line JSON output for every trial, including formatting failures if any
Analysis script	Bradley-Terry implementation, regularization setting, bootstrap procedure, random seed for bootstrap if used, and plotting code

Base hardness prompt template:

Quality-assurance forced-choice trial. Compare A and B as same-sized solid materials. Hardness means resistance to indentation and scratching. Exclude brittleness, fragility, size, weight, emotional valence, moral desirability, and clinical importance. Trial_id: [TRIAL_ID]. A: [ITEM_A]. B: [ITEM_B]. Choose the harder item. Return exactly one line of JSON and no other text: {"trial_id":"[TRIAL_ID]","choice":"A" or "B","confidence":[integer 0-100]}.

Wrapper variants should preserve the comparison definition, item labels, and JSON schema. Substantive prompt-family variants should be explicitly labeled as a new panel version.
